# An examination of service user satisfaction in forensic mental health settings

**DOI:** 10.1177/00258024241227719

**Published:** 2024-01-31

**Authors:** Al Adiya Khan, Victoria Stirrup, Douglas MacInnes

**Affiliations:** 1 Forensic and Offender Healthcare Services, 8956 Oxleas NHS Foundation Trust, Dartford, Kent, UK; 2 Department of Research Development, 2238 Canterbury Christ Church University, Canterbury, UK; 3 Faculty of Medicine, Health and Social Care, 2238 Canterbury Christ Church University, Canterbury, UK

**Keywords:** Finance, forensic psychiatry, rehabilitation, safety, satisfaction, staff interaction

## Abstract

High levels of service user satisfaction are viewed as a reliable indicator of a service providing good care and treatment. There has been limited research looking into levels of satisfaction in forensic mental health settings with most work focused on staff satisfaction in these settings. This study examined service users’ levels of satisfaction with a forensic mental health service in the United Kingdom. The service covered two sites; one a purpose-built secure unit and the other based in an old cottage hospital. Thirty-nine in-patients completed a 60-item validated forensic satisfaction scale. The scale measured seven domains of satisfaction as well as reporting an overall satisfaction score. The results indicated the service users were reasonably satisfied with the care and treatment they received. The domains of rehabilitation, safety, staff interaction and overall care showed the highest level of satisfaction. The high rehabilitation satisfaction score demonstrated the importance of meaningful activities for users accessing forensic services and may have been influenced by the security measures on the wards. The high safety domain score indicated respondents felt safe and secure within the wards and were likely to be influenced by positive interpersonal interactions. Good staff interaction was also an important factor in helping service users feel safe on the wards. These interactions are likely to be associated with longer periods of admission in secure services allowing therapeutic relationships to develop. Financial advice/support was the one domain that recorded negative satisfaction levels. Financial literacy training may help develop money management skills.

## Background

Service user satisfaction is an important concept to consider when examining mental health care.^1^ Satisfied patients are more likely to engage with services and adhere to therapy while dissatisfied patients are at higher risk of dropping out of treatment, and more likely to be treated in more restrictive settings.^2,3^ Satisfaction appears to be influenced by different factors such as patients’ expectation of services, the perception of the need for psychiatric care, the patient–clinician relationship, prior experience with services and the treatment received.^3–5^ High satisfaction ratings are seen as an indicator of good service organisation and delivery.

Satisfaction research within forensic mental health settings has focused on staff as participants.^6^ Reviews of quantitative studies examining service user satisfaction in forensic settings have concluded the veracity of the findings was questionable due to limited methodological rigour.^7,8^

Recent developments have seen validated new measures designed specifically to assess user satisfaction in forensic settings. A recent comprehensive systematic review of scales measuring service user satisfaction in mental health settings identified five forensic mental health satisfaction measures as able to present valid findings.^1^ This has enabled a greater exploration of service user satisfaction with research noting the positive correlation between user satisfaction and good therapeutic relationships.^9,10^

However, there has been a limited examination of the impact of demographic or environmental factors on levels of user satisfaction with forensic mental health services. This study aimed to examine the levels of service user satisfaction in forensic mental health in-patient units within a National Health Service (NHS) Mental Health Trust in England.

## Methods

### Design

A survey design was used.^11^ Data was collected with regards to service users’ satisfaction with the service provided to them at one time point.

### Participants

All service users cared for and treated as in-patients in one London forensic service were eligible for inclusion. Service users resided in medium and low-secure wards^12^ which were categorised as providing acute care or rehabilitation. The service is on two sites; Site A – a purpose-built 85-bed unit providing medium and low secure care for men and women and Site B – a 30-bed low secure unit for men on the grounds of an old cottage hospital.

Every current in-patient was eligible to participate with the only exclusion criterion being any service user who the clinical team determined was unable to participate in the study was not approached.

### Data collection

#### Demographic information

Information was collected about the following: age, gender, type of ward, ethnicity and length of time in the ward/unit. Age and length of time in the ward/unit were recorded in discrete ranges to help anonymise each respondent.

#### Satisfaction

Data on levels of satisfaction were collected via the validated self-report questionnaire, the Forensic Satisfaction Scale (FSS).^13^ It was viewed as valid by Miglietta et al. and has good concurrent validity with other scales.^1,14^ It is a 60-item measure with respondents rating their satisfaction with services on a 5-point Likert scale. The scale records levels of satisfaction in seven domains (staff interaction, rehabilitation, communication, milieu, finance, safety, and overall care) as well as gives a total score. Mean scores are calculated, with a possible range of one to five. Higher scores indicate greater levels of satisfaction. Project information sheets were given to all prospective participants. A researcher independent of the clinical team then met with potential participants at ward community meetings and answered any questions about the study prior to obtaining informed consent. The researcher handed out the questionnaires which, once completed, were placed in unmarked sealed envelopes and returned to the researcher. Respondents were offered help in completing the questionnaires if they had difficulties in reading or understanding the questions. Initially, service users were going to be invited to take part in focus groups to explore the findings of the survey, but these were prevented from taking place due to the COVID-19 pandemic.

#### Analysis

Descriptive data were reported regarding the demographic details and mean FSS scores on the seven domains as well as the total score.

Independent t-tests were undertaken to examine the differences in FSS mean scores between respondents in relation to a range of demographic characteristics, and organisational differences of the service.

#### Ethics

The project obtained ethics approval at Canterbury Christ Church University and from the Research and Development department at the NHS Foundation Trust where the service was based (Ref: 882).

## Results

At the time of the survey, there were 115 service users residing at the two sites. The clinical team advised it was inappropriate for 20 potential participants to be involved in the study due to concerns regarding aggressive behaviours or their ability to understand the information provided and inability to give informed consent. This left 95 potentially eligible respondents. Out of these, 14 potential participants (14.7%) did not attend any of the ward meetings and seven questionnaires (7.4%) were incomplete and unable to be included in the analysis. From the remaining users, 39 participants (52.7%) completed the questionnaire. Twenty-nine self-identified as male and 10 as female. The majority 27 (69%) were aged between 25 and 45 with six aged between 18 and 25 and only one in the 56+ age range. Nineteen stated they were white and 20 self-identified as Black or Asian. The amount of time they had been in the unit ranged from 10 who had been admitted between 0 and 6 months and six who had been in the unit for over three years. 29 participants responded from Site A and 10 participants from Site B. Nineteen respondents were in-patients in acute wards and 20 in rehabilitation wards.

Table 1 shows the scores for each of the domains and the total satisfaction scores.

**Table 1.** Forensic satisfaction scale scores.

	Communication mean (sd)	Finance mean (sd)	Milieu mean (sd)	Overall care mean (sd)	Rehabilitation mean (sd)	Safety mean (sd)	Staff interaction mean (sd)	Total mean (sd)
(N = 39)	3.21 (0.85)	2.97 (0.76)	3.22 (0.61)	3.54 (1.37)	3.58 (0.73)	3.56 (0.98)	3.52 (0.74)	3.39 (0.59)

The total satisfaction score of 3.39 is above the mid-point score of three indicating a reasonable degree of satisfaction with the service provided. Higher scores (above 3.5) are recorded for four domains (rehabilitation, safety, overall care and staff interaction) while one domain (finance) has a mean score below the mid-point score of 3.

The total satisfaction scores for demographic and organisational characteristics are noted in Table 2 together with the t-test scores that examined the differences between the reported outcomes.

**Table 2.** Demographic and organisational comparisons.

Variable	FSS total score (sd)	t test score (df)	*p*-value
**Age**			
18–35 (*n* = 19)	3.41 (0.47)	0.19 (37)	.85
36+ (*n* = 20)	3.38 (0.70)		
**Gender**			
Women (*n* = 10)	3.21 (0.52)	−1.17 (37)	.25
Men (*n* = 29)	3.46 (0.61)		
**Ethnicity**			
Nonwhite (*n* = 20)	3.39 (0.66)	−0.07 (37)	.94
White (*n* = 19)	3.40 (0.52)		
**Length of stay**			
≤ 2 years (*n* = 21)	3.40 (0.55)	0.02 (37)	.99
2 years+ (*n* = 18)	3.39 (0.65)		
**Ward type**			
Acute (*n* = 19)	3.38 (0.59)	−0.17 (37)	.86
Rehabilitation (*n* = 20)	3.41 (0.60)		
**Site**			
Site A (*n* = 29)	3.32 (0.61)	−1.33 (37)	.19
Site B (*n* = 10)	3.61 (0.51)		

All of the groups scored above the total mid-point satisfaction score of 3 suggesting a degree of satisfaction with the service. Most of the variables (age, ethnicity, length of stay, and ward type) showed small differences between different categories in total satisfaction scores. Although not statistically significant, there were clear differences in some scores with men scoring higher total satisfaction scores than women and those in Site B recording higher scores than the respondents from Site A. Post hoc analysis was then undertaken to examine differences in these scores. Significant differences were noted with Site B scoring significantly higher than Site A in the rehabilitation domain (Site A 3.39 (0.72) – Site B 4.12 (0.44), t−3.02 df(37), *p* = .01) and the overall care domain (Site A 3.28 (1.46) – Site B 4.30 (0.67), t−2.97 df(33.49), *p* = .01). No significant differences were reported between men and women.

## Discussion

The results indicate service users were satisfied with the care and treatment received. This is in keeping with other literature noting general satisfaction with forensic services.^9,15^ All domains recorded scores above the mid-point score of 3 except for help/support with finances. Four scores were above 3.5 (rehabilitation, safety, overall care and staff interaction) indicating a fair degree of satisfaction with their care. The domain of overall care normally records a high score as the questions ask for users’ overall views of the service and respondents often give inflated satisfaction responses. However, it is useful to examine the potential reasons for the high scores in the three other domains and the finance domain, which scored below the mid-point, as these focus on specific elements of care and treatment.

### Rehabilitation

The highest scoring domain was that of rehabilitation indicating respondents were happy with their rehabilitation opportunities. There is conflicting evidence concerning service users’ rehabilitation experiences in forensic settings with some studies reporting most users valued and enjoyed their occupational time^16^ while others found life on forensic wards monotonous with little to do.^17^ Most activities were self-initiated and forensic in-patients more likely to engage in activities in the late afternoon and evening which may explain the high level of self-initiated activities as these times were outside the normal hours worked by rehabilitation staff.^16^ Levels of satisfaction were higher when the focus of engagement was targeted on achieving positive goals rather than symptoms of mental illness and reducing offending^18,19^ suggesting user satisfaction increases when there are appropriate or meaningful interventions.

Participants residing in Site B reported significantly higher levels of satisfaction compared to those in Site A. It should be noted both sites scored well above the mid-point score with the very high levels of satisfaction recorded by Site B participants leading to these significant differences. Interestingly, there was no significant difference recorded between service users residing in medium as opposed to low security. Higher levels of security have previously been associated with lower quality of life scores.^20,21^ This is partly due to the forensic environment constraining how people choose, organise and enact rehabilitation activities.^22,23^ Increased environmental security measures also present fewer opportunities for engaging in meaningful activities, practising skills, and making independent decisions as well as limiting access to the wider community leading to lower levels of satisfaction.^21^ This may partially explain this finding between the difference in satisfaction between these sites with Site B not having many of the environmental features of a medium secure facility leading to a greater variety of rehabilitation opportunities. It has also been reported therapeutic skills of staff were higher when there was less of a focus on security.^24^ In-patients in more secure conditions were also more likely to have a poorer treatment response thereby reducing the opportunities for staff to gain skills and experience in rehabilitative activities. Staff in low-secure settings also viewed their therapeutic practices as being more valued which could also lead to them being more active in developing opportunities for therapeutic interventions.

### Safety

The safety domain recorded the second highest levels of user satisfaction suggesting the service had maintained a safe environment. Historically, user's perspectives of their safety in forensic settings have not received much attention with the focus being more on the safety of staff.^25^ Service users have reported feeling less safe when a peer's behaviour was escalating and threatening to staff or other users with this potentially leading to a further escalation of aggression.^26^ Aggressive behaviours have traditionally been managed through a combination of medication, restraint and seclusion. However, the emphasis has become more focused on using positive communication and prevention strategies. The importance of staff interactions will be discussed further in the next section but, in the context of safety, staff–user relationships have been identified as central facilitators in forensic mental health settings,^27^ with negative interactions identified as responsible for 2 out of every 5 violent incidents.^28^ Further, positive engagement on wards as well as increased levels of perceived support have been found to increase positive user-to-user interactions reducing conflict between service users.^29^ Service users have reported that a culture of care in the ward, especially positive communications with nurses, had the most significant impact on their perceptions of being safe^30^ while staff members perceived better communications and positive relationships with service users increased their safety on the unit.^31^

Longer periods of in-patient admissions in forensic services have also been proposed as a reason for higher levels of satisfaction with regard to personal safety.^32^ The longer periods of admission enable staff members to become more familiar with individual service users’ behaviours. It has also been noted that some staff members get along better with some service users than others.^26^ This can help in the development of effective approaches for de-escalating potentially violent incidents and supports staff members, who have a good rapport with an individual, taking the lead in de-escalating unsafe behaviour.

### Staff interaction

The staff interaction satisfaction scores also indicated positive relationships between staff and service users in the service. Positive interactions have been found to reduce frustration and increase overall satisfaction with services^6^ with the strongest association with user satisfaction being a good therapeutic relationship with staff.^9^ It is proposed this is because the relational aspect of care builds up trust and, through this, staff create a sense of security that contributes to recovery.^33^ Users who had problems with staff reported the lowest levels of satisfaction.^34^ One reason that the staff–user relationship in forensic settings is often strong is because many service users had been in forensic settings for several years and this allowed service users the time to develop a strong relationship with at least one staff member.^6^ Satisfaction with staff interactions has also been found to be higher when staff worked collaboratively with users and recognised the need for service users to have an element of choice and control over their lives.^17^ High staff-user ratios mean that staff often have the time and resources to work with service users allowing the opportunity to develop a positive therapeutic relationship with staff also available to quickly respond to users’ needs.^10,35^ It has also been found users become frustrated with staff members perceived as being disinterested or not spending time with them.^6^ Restricting users’ autonomy and their routines can negatively affect the relationship between staff and service users.^21^ As such, services should consider the impact of restrictive interventions from both an emotional and relational perspective.^36^

### Finance

The one score below the mid-point related to satisfaction with financial support. This has been rarely discussed in relation to forensic settings even though it is part of someone's personal and social identity.^37^ The impact of financial distress on mental health in general has been noted. Being poor increases the likelihood that a person will have a mental illness, exacerbates psychiatric symptoms, and increases the likelihood of poor general medical health.^38^ Those receiving disability or unemployment benefits have poorer mental health regardless of their personal, social and financial circumstances.^39^ Additionally, individuals with daily financial insecurity have reported feeling lonely and isolated and were unable to achieve important life goals.^40^ Living in poverty has a greater negative impact on the quality of life and functioning among individuals with serious mental illnesses than on any other groups in society.^41^ Financial instability was also associated with a profound sense of shame, low self-esteem, low self-efficacy, self-loathing, depression and suicidal thoughts and linked to an increased risk of relapse.^42^

However, there has been no discussion about financial insecurity in relation to forensic in-patient care. The lower level of financial satisfaction clearly indicates some unhappiness with the financial support and advice received by these forensic service users. In non-forensic settings, it is acknowledged there have been limited opportunities for teaching money management skills to service users.^42^ This has led authors to advocate interventions designed to develop these skills through financial literacy training with the goal of helping participants manage their own money.^38,41^ The training could include; educating people about how to budget and manage money, avoid predatory financial situations, reduce accumulated debt, and cope with limited financial resources.^43^

### Limitations

Although the study reports on a range of service user satisfaction domains in forensic in-patient settings, it is focused on a small sample from one service and is also cross-sectional. It would be helpful for future research to include a larger sample size from several forensic in-patient services using a longitudinal design.

A proportion of patients were ineligible to take part in the study and a proportion chose not to. It is difficult to say whether these groups constituted the more unsatisfied patient group or not though it is likely the non-participant group would have had a more disengaged or hostile relationship with the service.

Future studies using qualitative techniques would be helpful to both get further information about why different domains are rated high or low and to identify what may help increase satisfaction.

## Conclusion

This cross-sectional survey of 39 in-patients in one forensic service in England, indicated general satisfaction with the service received. There were few differences in levels of satisfaction recorded between demographic groups. The domains of rehabilitation, staff safety and staff interaction showed the highest level of satisfaction. These findings acknowledged the importance of meaningful activities for users accessing forensic services. Users perceived themselves to be safe and secure in the in-patient wards and influenced by positive interpersonal interactions between staff and service users. The extended time forensic service users are inpatients allows therapeutic relationships to develop. It was also acknowledged the security measures adopted by the forensic service impacted levels of satisfaction and conflicts surrounding these restrictions could compromise staff–user relationships. Financial advice/support was the one domain that scored below the midpoint score and therefore recorded negative satisfaction levels. Introducing financial literacy training may help service users develop money management skills and increase their satisfaction with the financial advice and support provided.
